# Microglial MS4A4A elevates TREM2 signaling and reduces amyloid pathology

**DOI:** 10.1002/alz70855_106607

**Published:** 2025-12-24

**Authors:** Xiao‐Fen Chen, Hengjun Rao

**Affiliations:** ^1^ Fujian Provincial Key Laboratory of Neurodegenerative Disease and Aging Research, Institute of Neuroscience, School of Medicine, Xiamen University, Xiamen, Fujian, China; ^2^ Shenzhen Research Institute of Xiamen University, Shenzhen, China

## Abstract

**Background:**

Triggering receptor expressed on myeloid cells 2 (TREM2) is a gene specifically expressed in brain microglia, with genetic variants associated with increased Alzheimer's disease (AD) risk. In addition to its membrane‐bound form, TREM2 is also released as a soluble form (sTREM2), which has been linked to improved cognitive outcomes in AD. Recent studies have identified MS4A4A as a key regulator of sTREM2; however, its precise role in regulating sTREM2 and its impact on AD pathology remain unclear.

**Method:**

To investigate this, we utilized the 5xFAD mouse model and overexpressed either wild‐type MS4A4A or the AD‐associated variant MS4A4A‐M159V specifically in microglia. We measured TREM2 and sTREM2 levels, evaluated microglial survival, and examined the clustering of microglia around amyloid plaques. Additionally, we assessed amyloid‐beta clearance, quantified amyloid burden, and evaluated cognitive function.

**Result:**

Overexpression of wild‐type MS4A4A resulted in significantly increased TREM2 and sTREM2 levels, promoted microglial survival, facilitated microglial clustering around amyloid plaques, and enhanced Aβ clearance. These changes led to a reduction in amyloid burden and improved cognitive function. In contrast, the MS4A4A‐M159V variant failed to produce these effects, and similar outcomes were observed in TREM2‐deficient models.

**Conclusion:**

Our findings underscore the critical role of MS4A4A in modulating AD pathology through TREM2. These results position MS4A4A as a promising therapeutic target for AD.

